# Alleviating Neonatal Intensive Care Unit Stress: A Chinese Medicine Approach in Neonatal Rats

**DOI:** 10.1155/2024/2733884

**Published:** 2024-03-02

**Authors:** Yingxin Li, Xi Huang, Yanlin Hu, Liming Yang, Xiujuan Zhang, Qiong Chen

**Affiliations:** ^1^Department of Neonatology Nursing, West China Second University Hospital, Sichuan University, Chengdu, Sichuan, China; ^2^Key Laboratory of Birth Defects and Related Diseases of Women and Children, Ministry of Education, Chengdu, Sichuan, China

## Abstract

**Background:**

Premature infants are exposed to numerous stressors in neonatal intensive care unit (NICU) during a crucial period for brain development; this period exerts long-term influences on cognitive and behavioral development.

**Aims:**

To evaluate the effect of NICU-related stress on neonatal rat pups and explore the effect of Chinese medicine treatment (CMT).

**Methods:**

Sixty male rat pups were randomly assigned to three groups: the control group, the NICU group (NICU-related stress), and the CMT group (NICU-related stress plus CMT). All stressors and interventions were administered from 0 to 7 days after birth. Body weight, serum corticosterone levels, and behavior in the open field (OF) test, elevated plus maze (EPM) test, sucrose preference test, and Morris water maze (MWM) test were recorded, and blood samples were collected at five different time points (T0, T1, T2, T3, and T4).

**Results:**

The body weights of rats in the CMT and control groups were heavier than those in the NICU group in both early life and adulthood (*P* < 0.05). Serum corticosterone levels significantly differed with time (except T0 vs. T1 and T3 vs. T4) but did not significantly differ among the three groups (*F* = 0.441, *P* = 0.894). Regardless of age, spatial memory and anxiety-like and depression-like behavior did not differ among the three groups.

**Conclusion:**

NICU-related stress exerted a long-term effect on rat growth and development but did not affect spatial memory, anxiety-like behavior, depression-like behavior, or serum corticosterone levels. CMT alleviated the impact of NICU-related stress on rats and promoted the growth and development of neonatal rats.

## 1. Introduction

Every year, an estimated 15 million premature infants are born worldwide. Preterm birth rates constitute 5% to 18% of live births in 184 countries, and the two countries with the most preterm births are India and China [1]. Preterm birth is a major health issue for infants [2]. Because their organs are not fully developed, most of these neonates must stay in the NICU for several weeks [3]. Although advances in the NICU care have markedly improved the survival rate of premature infants, they are exposed to various NICU stressors, such as high levels of sound and light, repeated pain stimuli, and maternal separation, during a crucial stage for brain development, resulting in unfavorable outcomes [4–6]. These stressors have profoundly influence pain sensitivity and emotional, cognitive, and behavioral development as well as the programming of hypothalamic–pituitary–adrenal (HPA) axis [4, 7–13]. Early-life stress has been linked to long-term changes in the brain and behavior [14, 15]. Montirosso et al. found that NICU-related stress might be related to temperamental difficulties at 3 months of age in premature newborns and decreases in brain size at term equivalent postmenstrual age [5].

In addition, repeated exposure to pain as a neonate has been related to impaired development of the corticospinal tract of premature babies and decreased head circumference [4, 16, 17]. Ranger et al. showed that procedure-related pain in neonates was related to a thinner cortex at school age [18]. Vinall et al. reported that increased exposure to repeated pain in premature newborns is related to a lower IQ [19]. Chen et al. found that repeated exposure to pain led to impairments in spatial learning and increases in anxiety-like behavior in rats [3].

Interestingly, research on the impact of NICU-related stress on neonates exhibits considerable inconsistency. In contrast, Mooney-Leber and Brummelte showed that pain stimulation promoted spatial learning of the platform location in the Morris water maze [10]. Pryce et al. demonstrated that exposure to early-life stress improved cognitive function [20, 21]. Additionally, other studies have reported that compared with control animals, animals exposed to pain did not differ in HPA axis reactivity [22, 23]. Given these inconsistencies in the current studies, further research is required to confirm the impact of NICU-related stress on preterm infants.

Because of the plasticity and fragility of the developing central nervous system of newborns, environmental factors play an essential role in brain development. Exposure to negative environmental stimuli, such as malnutrition, deprivation, pain, and disease, in early life, can impair intellectual development; conversely, appropriate enrichment of environmental stimuli can enhance impaired synaptic plasticity [24].

Chinese medicine treatment (CMT) is based on traditional Chinese medicine's (TCM) theory that balance is beneficial. This high-level generalization describes the relationship among the human body, the environment, and diseases. CMT nursing methods (including acupuncture massage, environmental nursing, and the five elements of music) are used for treating and preventing disease [25]. Additionally, CMT nursing methods are effective for treating stress-related diseases. Nevertheless, the effect of CMT on neonates subjected to NICU-related stress is unknown.

Thus, we aimed to determine whether CMT was an effective treatment for neonatal rats subjected to NICU-related stress. We hypothesized that (1) NICU-related stress in rats would lead to impaired spatial learning as well as increased anxiety-like and depression-like behavior, (2) NICU-related stress in rats would result in altered stress responses, and (3) CMT would reduce the impact of NICU-related stress. First, we observed spatial learning and changes in behaviors in both prepubescent and adult rats exposed to NICU-related stress in the neonatal period. Second, we examined changes in serum corticosterone levels in both prepubescent and adult rats during the stress response. Finally, we determined the effect of CMT on rats subjected to NICU-related stress in the neonatal period.

## 2. Materials and Methods

### 2.1. Experimental Animals

Our study was performed in strict compliance with the recommendations of the National Research Council's Guide for the Care and Use of Laboratory Animals. Our protocol was approved by the Ethical Committee of West China Second University Hospital, Sichuan University (permit number: 2021, Animal Ethics Approval No.: 014). Every effort was made to minimize rat suffering and to reduce the number of rats used.

Sixty male Sprague–Dawley rats (Chengdu Dossy Experimental Animals Co., Ltd., China) were purchased on postnatal day 0 (P0). They were housed in the experimental animal center affiliated with West China Second University Hospital under controlled environmental conditions: temperature (20~26°C), humidity (60~65%), and a set light cycle (lights on: 8:00 to 20:00, lights off: 20:00 to 8:00). Every dam had 10 pups in their litter. The rat pups were randomly divided into three groups, and each group was then assigned to different dams in a random order. All litters were undisturbed after the experimental manipulation, except for routine changes in bedding. The pups were weaned on P21, and four weaned rats were kept in each cage until further tests. The weaned rats were fed the same food.

### 2.2. Experimental Design

**Figure 1:**
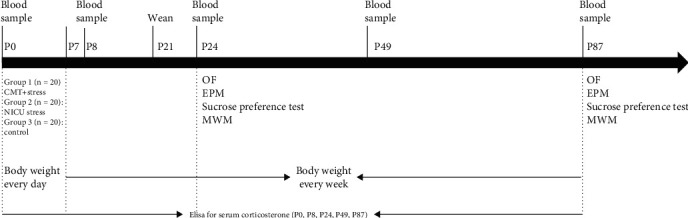
The experimental timeline. On the day of birth (postnatal day 0 (P0)), male rats were randomly assigned to groups; NICU-related stressors and CMT intervention were administered from P0 to P7. Then, all rats were left undisturbed until weaning at P21. The CMT group received environmental enrichment from P21 to P49. Blood samples and ELISA to assess serum corticosterone levels were performed on P0, P8, P24, P49, and P87. On P24-P35 and P87-P98, rats underwent the open field (OF), elevated plus maze (EPM), sucrose preference, and Morris water maze (MWM) tests. Body weight was measured every day during the neonatal period from P0 to P7 and weekly thereafter.

This experiment utilized a randomized controlled trial (RCT) design. Sixty male Sprague–Dawley rats were randomly assigned to three groups of twenty rats each using a random number table at P0. We decided on the sample size by the literature [3]. The CMT group received NICU-related stress plus CMT, the NICU group was subjected to NICU-related stress, and the control group did not receive any stressors or interventions. In addition, all NICU-related stressors and interventions were applied from P0 to P7. The experimental timeline is shown in Figure 1.

### 2.3. Experimental Procedures

#### 2.3.1. NICU-Related Stress

*(1) Repeated Exposure to Pain*. Repeated neonatal pain was induced similar to that in Chen et al.'s study [3]. The pain exposure for the CMT and NICU groups was as follows: one of each rat pup's paws received four needle sticks every day from P0 to P7. The needle stick was performed automatically via a blood glucose sampling device (26G XY2, MEDSUN, Ningbo, China). To prevent cross-infection, each needle was used only once. The needle was quickly inserted into the middle of the foot; afterwards, a cotton ball was used to stop the bleeding. The rats were then put back in their cage. The stressor was applied to the CMT group and the NICU group from the day of birth (P0) to the seventh day after delivery (P7).

*(2) Noise*. Noise was provided by playing a recording of sound from an actual NICU, including the sounds of monitor alarms, ventilators, and other equipment; medical staff talking; opening and closing the incubator door; and the treatment cart rolling across the floor. The sound level was set at 90 dB. The noise intervention was conducted simultaneously with the exposure to repeated pain, from P0 to P7. Each rat pup received four noise interventions per day, each lasting one hour.

*(3) Light*. We applied fluorescent lamp irradiation (150 lx) for 4 one-hour sessions each a day from P0 to P7.

#### 2.3.2. Chinese Medicine Treatment

*(1) Acupuncture Massage*. The selection of acupuncture points was in accordance with the rat acupuncture points presented in the *Handbook of Practical Animal Acupuncture* [26]. Our selection of acupuncture points is primarily based on the preterm developmental care theory and the functions of the acupuncture points. Optimizing nutrition, minimizing pain, and safeguarding sleep are important components of developmental care for preterm infants. Therefore, we selected acupuncture points that alleviate pain, improve sleep, and enhance gastrointestinal function to mitigate NICU-related stress. The chosen acupuncture points included *Hoku* (between the 1st and 2nd metacarpal bones of the forelimbs, one point on each side), *Zusanli* (below the knee joint, in the muscle groove at 0.5 cm below the small head of the fibula, one point on each side), *Shenmen* (the edge on the ulna bone of the wrist striation on the medial forelimb, one acupoint on each side to regulate cranial nerve function), and *Sanyinjiao* (1 cm above the inner tip of the ankle of the hind limb, one point on each side). We used a modified cotton swab to apply downward pressure with the cotton tip; gentle wrist movements were used to apply gentle pressure at regular intervals, 80~120 times/min. The acupuncture massage began 1 minute before the needle stick and lasted approximately 1-2 minutes per point. Each massage session took approximately 20 minutes and occurred four times per day from P0 to P7.

*(2) Environmental Enrichment*. Referring to the Chinese researcher Zhang, a Youyu-like TCM environment was created in the cages of the CMT group, consisting of dark, curved passages, wooden ladders, teeth-grinding items, etc. [25].

*(3) Five Elements of Music*. The music recording “Five Elements of Music” was created by China Medical Audiovisual Publishing House, composed by Shi Feng, and performed by the Central Conservatory of Music Folk Orchestra. This recording was played four times a day for 30 minutes each. The distance between the rat cage and the sound box was kept at 0.5 m. The music was played in a quiet environment to prevent sound interference from other sounds, and the sound level of the music was set at 40 dB.

#### 2.3.3. Body Weight

Body weight was recorded at 20 different time points. It was measured daily in the neonatal period (P0-P8) and weekly thereafter (P14, P21, P28, P35, P42, P49, P56, P63, P70, P77, P84, and P91).

#### 2.3.4. Morris Water Maze (MWM) Test

The MWM test was used to assess spatial learning and memory in rats. It is a widely used behavioral test in neuroscience and psychology for assessing spatial learning and memory in rodents. A total of 10 rats were tested in each group at each time point. A blue water tank measuring 160 cm in diameter, 50 cm in height, and 30 cm in depth was used. During the test, the temperature of the water in the tank was maintained at 23 ± 2°C. Four marked reference points were evenly distributed around the circumference of the tank, thus dividing the tank into quadrants (southeast (SE), southwest (SW), northeast (NE), and northwest (NW)). A black platform (10 cm in diameter) was placed 2 cm below the water surface in the SW quadrant. The wall of the tank was marked with a wealth of reference cues (such as rectangles, squares, triangles, and circles) to assist rats in locating the platform. The entire MWM test included a training phase (days 1-5 (D1~D5)) and a probe trial (D6). It took six days to complete the whole experiment. In the training trials, the platform was placed beneath the surface of the water, and all rats underwent four trials a day for five consecutive days. In each trial, rats were randomly placed in the water facing the wall of the tank in one of the four quadrants (SE, SW, NE, or NW). All rats that found arrived the platform were allowed to stay on it for 10 seconds. If the rats did not find the platform within 90 seconds, the experimenter guided them to its location.

During the training trials, latency (swim time from start to locating the platform) and distance (swim distance from start to locating the platform) were recorded. The rats underwent a probe trial 24 hours after the end of the training phase to evaluate their spatial memory. At the last day of test, the platform was removed. The measurement indexes included latency, the crossing index (number of platform site crossings), swim duration in the target quadrant (SW), and swim duration in the opposite quadrant (NE). After training, rats were dried with tissue and an infrared heating lamp when the training was finished and then returned to their cages.

#### 2.3.5. Open Field (OF) Test

The OF test was used to examine anxiety-like and exploratory behavior. We used a square open field area (100 × 100 cm) consisting of an empty and bright square arena surrounded by four plastic walls that prevented the rats from escaping. Each rat was given 5 minutes of acclimation in the open field before the trial began. At the beginning of the trial, they were placed in the middle of the arena. Each rat's behavior was recorded for a duration of 10 minutes. The OF test area was fully cleaned with 75% alcohol after each rat finished the test, and subsequent rats were not tested until the alcohol odor had faded. Duration in the center region, duration in the peripheral region, distance traveled in the center region, distance traveled in the peripheral area, and total crossings (number of entries into the central or peripheral area) were measured.

#### 2.3.6. Elevated Plus Maze (EPM) Test

The EPM test was used to assess anxiety-like behavior. The EPM apparatus consisted of a “+”-shaped maze elevated above the ground, including a center area, two opposing closed arms (10 cm × 50 cm × 30 cm), and two opposing open arms (10 cm × 50 cm). Each rat was given 5 minutes to acclimate in the EPM before the test began. Rats were placed in the center area at the beginning of the trial, and their behavior was recorded for 5 minutes. The maze was scrubbed with 75% alcohol and dried after every session. Duration and distance in the different areas (open arms and closed arms) and the total number of arm entries were recorded.

#### 2.3.7. Sucrose Preference Test (SPT)

The SPT was used to assess depression-like behaviors. Each rat was placed in a separate cage to acclimate one day before the test. During the test, rats were provided with two water bottles, one containing pure water and the other containing a 1% sugar solution. The two bottles were placed on either side of the cage, with the sides randomly assigned to exclude the possibility of location preference. On the first day of the test, rats were given two bottles of sugar water to acclimate to the taste; on the second day, they received one bottle of sugar water and one bottle of purified water. On the third day, the rats were fasted for 24 hours and then presented with a bottle of sugar water and a bottle of purified water. Consumption of the sucrose water and purified water was recorded.

In all behavioral tests, the rats were recorded with an infrared camera system (SSC-CB561R, Sony, Japan). In addition, a behavioral video tracking system was used to label rat behaviors (SMART V3.0.02, RWD Life Science, China).

#### 2.3.8. Blood Sampling

Blood samples were collected at five different time points (T0, T1, T2, T3, and T4): at birth on P0 (T0), after they were subjected to NICU-related stressors on P8 (T1), at P24 (T2), at P49 (T3), and at P87 (T4). Samples were taken at the same time each day (8:00~12:00 am) to exclude the influence of time of day. On the sampling days, the rats were moved from the feeding center to the laboratory, where they were anesthetized with isoflurane in a glass chamber. After anesthesia, the rat was placed on a cushion to gather blood. Blood was collected by decollation on P0 and P8. On P24 (T2), P49 (T3), and P87 (T4), the caudal vein was punctured with a 2.5 ml needle to collect a blood sample. Blood was collected in a blood collection tube (3.5 ml, BD Vacutainer SST II Advance, Switzerland). The rats were monitored until they awakened and then returned to their cages. The blood was centrifuged at 1800 × *g* for 10 minutes. The serum was removed and stored at -80°C.

#### 2.3.9. ELISA to Determine Serum Corticosterone Levels

The corticosterone concentration in the serum was analyzed using a corticosterone ELISA kit (ab108821, Abcam, UK). The corticosterone level results are expressed in ng/ml.

### 2.4. Statistics

Descriptive analyses of these datasets were used to determine whether they were normally distributed. Repeated measures analysis of variance (ANOVA) was used to assess the differences in body weight and MWM data. One-way ANOVA was used to analyze the OF test and EPM test. Serum corticosterone levels were analyzed using a two-way ANOVA, with group and time as factors. *P* < 0.05 was accepted as significant. Statistical analyses were performed using SPSS 23.0 for Mac.

## 3. Results

### 3.1. Body Weight

**Figure 2:**
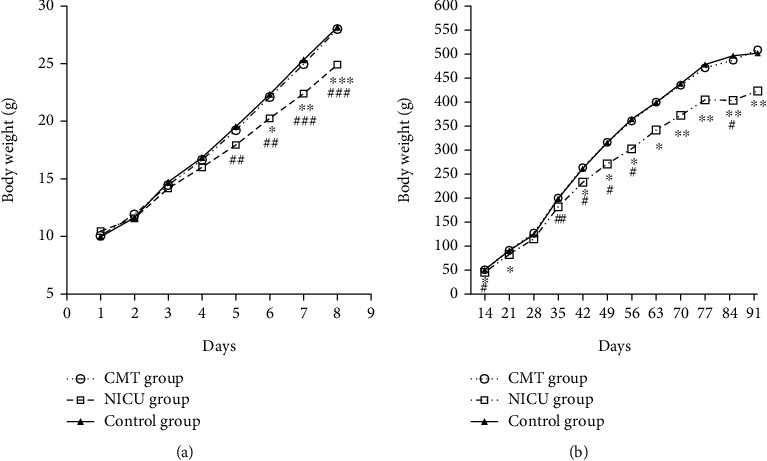
Body weight is expressed as the mean ± standard error of the mean (SEM). (a) The body weight in the neonatal period. (b) The body weight during P14-P91 period. ^∗^*P* < 0.05, ^∗∗^*P* < 0.01, and ^∗∗∗^*P* < 0.001 for the CMT group vs. the NICU group. ^#^*P* < 0.05, ^##^*P* < 0.01, and ^###^*P* < 0.001 for the control group vs. the NICU group.

In the neonatal period, the daily body weight of the rats in the CMT, the NICU, and the control groups increased with age, and there was a significant difference in the growth trend among the three groups (Figure 2(a), *F* = 5.735, *P* = 0.006). A least significance difference (LSD) method was used to evaluate the differences between the two groups. The difference between the CMT group and the NICU group (Figure 2(a), *P* = 0.008) and between the NICU group and control group (Figure 2(a), *P* = 0.004) was statistically significant. However, there was no difference between the CMT group and control group.

In the prepubescent and adult rats, the growth trends significantly differed among the three groups (Figure 2(b), *F* = 4.906, *P* = 0.016). There were significant differences between the CMT group and NICU group (*P* = 0.012) and between the NICU group and control (*P* = 0.011). However, there was no difference between the CMT group and control group.

### 3.2. Corticosterone Levels

**Figure 3:**
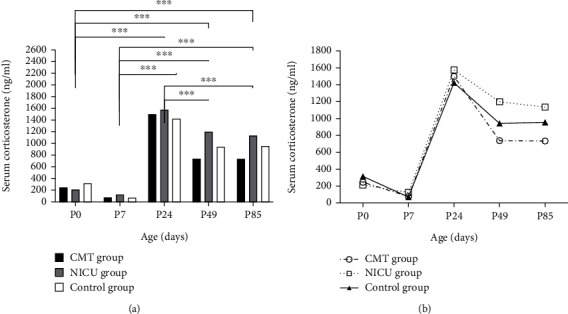
Serum corticosterone levels. Data are expressed as the mean ± SEM. ^∗∗∗^*P* < 0.001 for T0 vs. T2, T0 vs. T3, T0 vs. T4, T1 vs. T2, T1 vs. T3, T1 vs. T4, T2 vs. T3, and T2 vs.T4.

There was no time × group interaction (Figure 3, *F* = 0.441, *P* = 0.894). Additionally, there was not a significant main effect of group (Figure 3, *F* = 0.441, *P* = 0.894). There were no significant differences between T0 and T1, nor between T3 and T4. However, the differences among other time points were statistically significant. There was a decrease in serum corticosterone levels from P0 to P7; later, these levels peaked at P24 and plateaued at P49 and P85.

### 3.3. MWM Test

#### 3.3.1. Prepubescent Rats (P24-P30)

**Figure 4:**
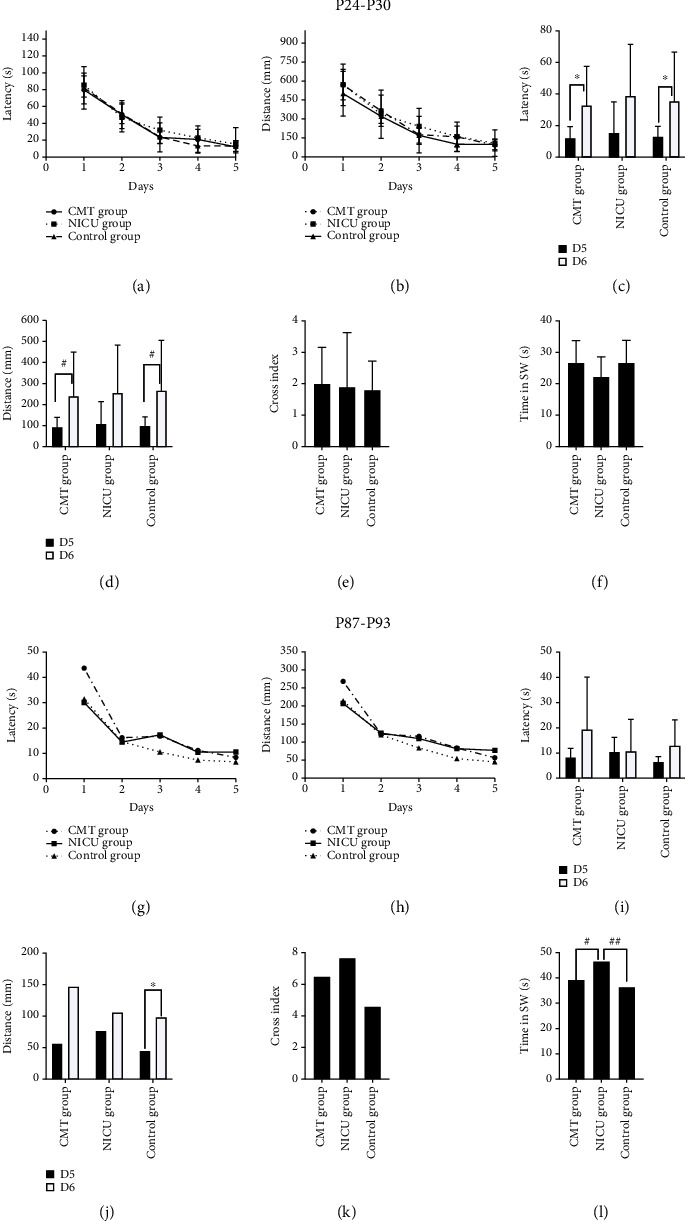
MWM results. ^∗^*P* < 0.05 for D5 (last day of the learning phase) vs. probe trial; ^#^*P* < 0.05 for the CMT group vs. the NICU group; ^##^*P* < 0.05 for the NICU group vs. the control group.

During the training trials, there were no group differences in latency to find the platform (Figure 4(a), *F* = 0.100, *P* = 0.905) or swimming distance before reaching the platform (Figure 4(b), *F* = 0.033, *P* = 0.967). During the probe trial, there were no group differences in the crossing index (Figure 4(e), *F* = 0.058, *P* = 0.944) or the duration in the SW quadrant (Figure 4(f), *F* = 1.418, *P* = 0.260). However, there was a significant difference in the latency to locate the platform between the last day of the training phase (D5) and the probe trial day (D6) in the CMT group (Figure 4(c), *t* = −2.570, *P* = 0.019) and control group (Figure 4(c), *t* = −2.250, *P* = 0.037). Similarly, there was a significant difference in the swim distance before locating the platform between D5 and D6 in the CMT group (Figure 4(d), *t* = 3.706, *P* = 0.002) and control group (Figure 4(d), *t* = 3.856, *P* = 0.001).

#### 3.3.2. Adult Rats (P87-P93)

In adult rats, no differences were observed in the time taken to locate the platform (Figure 4(g), *F* = 2.328, *P* = 0.116) or in the swimming distance required to reach the platform (Figure 4(h), *F* = 1.597, *P* = 0.220) among the three groups during the training phase. During the probe trial, the crossing index values also showed no significant differences (Figure 4(k), *F* = 1.232, *P* = 0.307). Moreover, there was no significant difference in the time to find the platform between the last day of the training phase (D5) and the probe trial day (D6) across the three groups (Figure 4(i), *F* = 2.238, *P* = 0.012). The control group exhibited a significant difference in the swim distance before reaching the platform location between D5 and D6 (Figure 4(j), *t* = −3.190, *P* = 0.011), but this difference was not found in the other groups. Regarding the duration in the SW quadrant, there were significant differences among the three groups (Figure 4(l), *F* = 5.373, *P* = 0.011); specifically, the NICU group spent more time in the SW quadrant than the CMT group (95% CI: 7.55, 13.93) and control group (95% CI: 3.61, 16.26).

### 3.4. OF Test

**Figure 5:**
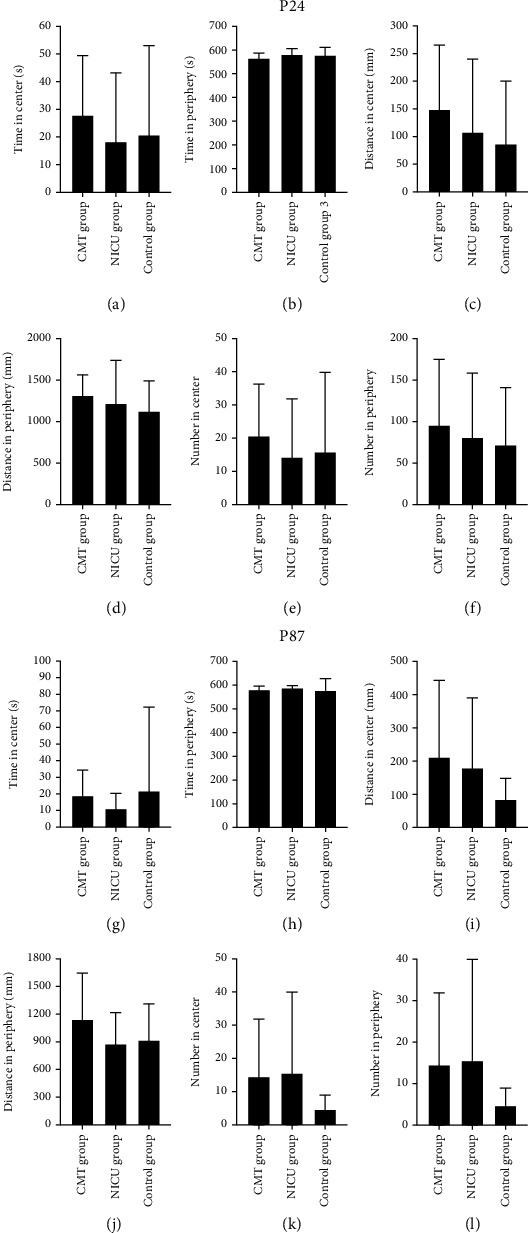
OF results.

Regardless of age, rats did not differ in the duration in the center area (P24: Figure 5(a), *F* = 0.349, *P* = 0.709; P87: Figure 5(g), *F* = 0.289, *P* = 0.751), duration in the periphery (P24: Figure 5(b), *F* = 0.956, *P* = 0.397; P87: Figure 5(h), *F* = 0.349, *P* = 0.709), distance traveled in the center (P24: Figure 5(c), *F* = 0.688, *P* = 0.511; P87: Figure 5(i), *F* = 1.301, *P* = 0.290), distance traveled in the periphery (P24: Figure 5(d), *F* = 0.575, *P* = 0.569; P87: Figure 5(j), *F* = 1.143, *P* = 0.334), number of entries to the central area (P24: Figure 5(e), *F* = 0.297, *P* = 0.745; P87: Figure 5(k), *F* = 1.190, *P* = 0.320), or number of entries to the peripheral area (P24: Figure 5(f), *F* = 0.250, *P* = 0.780; P87: Figure 5(l), *F* = 1.190, *P* = 0.320) among the three groups.

### 3.5. EPM Test

Regardless of age, rats did not differ in the duration in open arms (P24: *F* = 0.555, *P* = 0.581; P87: *F* = 0.270, *P* = 0.766), duration in closed arms (P24: *F* = 0.227, *P* = 0.798; P87: *F* = 0.089, *P* = 0.915), distance travelled in open arms (P24: *F* = 0.424, *P* = 0.659; P87: *F* = 0.648, *P* = 0.531), distance travelled in closed arms (P24: *F* = 0.12, *P* = 0.887; P87: *F* = 0.217, *P* = 0.807), number of entries to open arms (P24: *F* = 0.82, *P* = 0.922; P87: *F* = 0.417, *P* = 0.663), or number of entries to closed arms (P24: *F* = 0.594, *P* = 0.559; P87: *F* = 2.492, *P* = 0.102) among the three groups (Table 1).

### 3.6. Sucrose Preference Test (SPT)

There was no difference in sucrose preference (%) among the three groups in prepubescent (*F* = 0.098, *P* = 0.907) or adult rats (*F* = 0.101, *P* = 0.904).

## 4. Discussion

Because of the plasticity of the brain and its vulnerability of the brain during development, early life is a crucial period for brain development. NICU-related stressors, such as exposure to repeated pain, noise, light, and maternal separation, have long-term negative impacts on the brain development of newborns, resulting in mood and behavioral disorders. To date, many papers have examined the effect of repeated pain on neonatal rats. However, few have explored the effect of NICU-related stressors on neonatal rats. In this study, our results did not fully support our hypothesis; however, interestingly, we found that (1) NICU-related stress had a long-term effect on rat growth and development and (2) CMT could alleviate the effects of NICU-related stress in rats. Specifically, CMT promoted the growth and development of neonatal rats subjected to NICU-related stress. (3) However, NICU-related stress did not affect spatial memory, anxiety-like behavior, depression-like behavior, or serum corticosterone levels.

NICU-related stressors affect the growth and development of rats. We demonstrated that NICU-related stressors affected the growth and development of rats, reducing weight gain from neonatal life to adulthood. Specifically, we found that rats in the control group exhibited better growth trends than the NICU group, whether in neonatal life or adulthood. Similarly, Anand et al.'s findings are consistent with our results. Anand et al. found that rat pups that experienced a needle stick had significantly lower body weights on P8 and P15 compared to the control group [22]. Interestingly, other studies have found inconsistent results. Bernardi et al. found that neither the infliction of painful nor manipulation stimuli twice daily affected the body weight of the rats [27]. Walker et al. reported that repetitive pain did not change the body weight of the rats from P2 to P14 [23]. Mooney-Leber and Brummelte did not find differences in body weight gain during early life between rats in the stress exposure group and the control group [10]. Xia et al. found that there were no differences in weight gain between the needle stick group that experienced repetitive pain and the control group that did not [13]. Chen et al. reported that body weight gain was similar between the needle and the tactile groups [3]. This inconsistency in the literature regarding the effects of NICU-related stress exposure shows that changes in body weight may be dependent on the type, intensity, and duration of stress experienced. In the future, unifying the types of NICU stress stimuli, the intensity of the stimuli, and their duration and establishing standard operating procedures could be key to resolving these inconsistencies.

CMT promoted the growth and development of neonatal rats subjected to NICU-related stressors. Our results showed that the CMT group exhibited a better growth trend than the NICU group. Although no study has explored the benefits of CMT for neonatal rats experiencing NICU-related stress, a few studies have shown that CMT promotes neonatal growth and development. Jin et al. reported that massage promoted growth and development in neonatal infants and increased their ability to cope with stress [28]. The potential mechanisms are as follows: (1) acupoint massage on *Zusanli* may promote the secretion of hormones such as ghrelin and growth hormone (GH). Studies have shown that electroacupuncture at the *Zusanli* acupoint can enhance energy metabolism in rats by improving the pathological morphology of rat jejunum tissue, increasing the content of ghrelin, ATP, and cAMP in rat jejunum tissue, and upregulating PKA expression [29]. (2) Acupoint massage on *Shenmen* promotes sleep, and deep sleep is the strongest stimulant for the secretion of GH, so promoting sleep may indirectly affect the secretion of GH. (3) Five-element music therapy is a music therapy that combines music and TCM, based on the theories of “Yin-Yang and the Five Elements” and “Unity of Heaven and Man” with traditional Chinese musical instrument performance. It is divided into five modalities: Gong mode, Shang mode, Jue mode, Zhi mode, and Yu mode. In this study, the Gong mode and Yu mode were selected as the intervention music. Studies have shown that the Gong mode can strengthen the spleen and stomach, promoting the digestive and absorptive functions of preterm infants, playing a significant role in the weight gain of preterm infants [30]. This may explain why rats in the CMT group had a better body weight than those in the NICU group.

NICU-related stress did not affect the rats' spatial memory, anxiety-like behavior, depression-like behavior, or serum corticosterone levels. Our results suggested that NICU-related stressors did not influence rats' spatial memory, anxiety-like behavior, or depression-like behavior. We did not observe changes in anxiety-like behavior on the OF test or EPM test, nor any change in depression-like behavior in the SPT following NICU-related stress. We found no differences in spatial memory on the MWM test in prepubescent and adult rats. Similarly, Schellinck et al. showed that acute repeated pain exposure during the neonatal period did not change spatial learning in the MWM test [31]. However, our results are somewhat inconsistent with Chen et al.'s study. Chen et al. found that repetitive procedural pain led to impaired spatial learning in rats [3]. Regarding anxiety-like behavior, the literature is inconsistent. Anseloni et al. found that rats exposed to early inflammatory pain exhibited reduced depressive-like behavior compared to controls [32]. Similarly, Victoria et al. found that rats that experienced inflammatory pain early in the neonatal period displayed a decrease in anxiety-like behavior [33]. In contrast, Chen et al. and Anand et al. found that neonatal repetitive pain led to increased anxious-like behavior [3, 22]. Schellinck et al. also found that mice that experienced repeated needle stick pain early in life showed increased anxiety-like behavior during adolescence [31]. In addition, clinical studies showed that worse cognitive and motor functions were related to more skin-breaking procedures, and introverted behaviors such as anxiety, depression, and withdrawal increased at 18 months [34, 35]. These inconsistencies may be due to the fact that the intensity of stress exposure and the intensity of procedural pain may not be sufficient to affect spatial memory and anxiety behavior in adolescent and adult rats. Future studies should consider exploring the effects of stress of different intensities and exposure durations on the behavior of newborn rats. Additionally, in maternal care, behaviors such as licking by rat mothers after exposure to stress may mitigate the impact of stress. Future studies should exclude the mitigating effects of maternal care on stress responses.

Regarding the effect of NICU-related stress on the serum corticosterone levels of neonatal rat pups, we found that there was no difference among the three groups. In line with this finding, Chen et al. reported that there was no difference in serum corticosterone levels between the needle and control groups on P8 and P15 [3]. In contrast, Victoria et al. found a significant increase in corticosterone levels at 24 hours and 7 days after a carrageenan injection in newborn rat pups [36]. However, these levels decreased at 48 hours after exposure to the pain [36]. This inconsistency in the literature regarding the effects of NICU-related stress exposure suggests that changes in anxiety-like behavior may depend on the kinds or intensity of stressors experienced in the NICU. Considering the complexity of stress responses, oxytocin, GH, adrenocorticotropic hormone (ACTH), and brain-derived neurotrophic factor (BDNF) could be considered as biomarkers for future research. Additionally, the epigenetic changes in stress-related gene expression could also be studied.

## 5. Conclusions

We discovered that NICU-related stress has a lasting impact on the growth and development of rats, suggesting that future clinical scenarios should focus on enhancing the NICU environment to minimize stress in preterm infants. This could involve measures such as reducing pain, maternal separation, noise, and exposure to light. However, this stress did not influence their spatial memory, anxiety-like and depression-like behavior, or serum corticosterone levels. Larger studies may be required to validate these observations. CMT alleviated the effects of NICU-related stress and promoted the growth and development of neonatal rats. Maternal care might be a confounding variable, potentially weakening the effects of NICU stress and affecting the stress responses of newborn rats under NICU stress. Nonetheless, in our study, we endeavored to standardize maternal care conditions as much as possible across all groups. Furthermore, we also employed a cross-fostering strategy, allowing nonbiological mother rats to nurture all the pups, aiming to minimize the influence of behavioral differences between biological mothers and pups on the study outcomes. Moving forward, we plan to investigate and broaden the range of biochemical markers for assessing the effectiveness of CMT in NICU-related stress. Biomarkers for future studies could include oxytocin, GH, ACTH, and BDNF.

## Figures and Tables

**Table 1:** EPM results. Data are presented as the mean ± SEM.

Age	P24			P87		
Behavior/groups	CMT group	NICU group	Control group	CMT group	NICU group	Control group
Duration in open arms (s)	14.79 ± 5.76	10.81 ± 6.32	19.76 ± 5.99	28.03 ± 7.25	24.34 ± 8.73	32.13 ± 6.39
Duration in closed arms (s)	323.98 ± 39.16	355.73 ± 434.90	317.67 ± 45.10	230.30 ± 13.90	234.53 ± 15.87	226.71 ± 8.77
Distance traveled in open arms (mm)	44.72 ± 13.70	33.62 ± 10.96	29.91 ± 10.58	112.12 ± 12.67	108.84 ± 19.74	153.44 ± 47.16
Distance traveled in closed arms (mm)	389.12 ± 52.28	360.87 ± 32.24	393.95 ± 64.76	352.26 ± 41.28	331.64 ± 28.43	360.86 ± 21.57
Entries to open arms (n)	3.20 ± 1.12	2.90 ± 0.66	3.40 ± 0.79	4.70 ± 1.08	4.44 ± 0.84	6.00 ± 1.72
Entries to closed arms (n)	16.30 ± 7.10	9.50 ± 1.74	15.20 ± 3.71	16.90 ± 2.58	10.56 ± 1.47	14.00 ± 1.64

## Data Availability

We do not have a hyperlinks to publicly accessible archived datasets, but if needed, we can send the raw data to you.
